# The role of osteocalcin in regulating the acute stress response

**DOI:** 10.3389/fphar.2025.1646558

**Published:** 2025-07-14

**Authors:** Ning Kang, Jie Huang, Xiaoguang Han, Zhengqian Li, Yi Yuan, Xiangyang Guo, Ning Yang

**Affiliations:** ^1^ Department of Anesthesiology, Peking University Third Hospital, Beijing, China; ^2^ Department of Orthopedics, Peking University Third Hospital, Beijing, China; ^3^ Department of Spine Surgery, Beijing Jishuitan Hospital, Beijing, China; ^4^ Department of Spine Surgery, Peking University Fourth School of Clinical Medicine, Beijing, China; ^5^ Beijing Key Laboratory of Robotic Orthopaedics, Beijing, China; ^6^ Department of Anesthesiology, Beijing Jishuitan Hospital, Beijing, China; ^7^ Beijing Center of Quality Control and Improvement on Clinical Anesthesia, Beijing, China

**Keywords:** osteocalcin, acute stress response, HPA axis, anxiety and depression, cognition, bone-brain axis

## Abstract

Osteocalcin (OCN), a bone-derived hormone, considered as an indicator of bone turnover. Beyond its canonical role in bone metabolism, OCN may have many other functions as well. Studies have shown that it may also regulate glucose and lipid metabolism, cognitive function, sexual function, and more. Recently, OCN has become one interesting hormone with potential effects on acute stress response (ASR), which is essential for vertebrates’ survival. This review aims to comprehensively summarize the progress on the role of OCN in the pathophysiology of ASR and to thoroughly analyze the molecular mechanisms and significance of OCN in modulating ASR. In summary, a deeper understanding of OCN’s role in the ASR will help reveal how bone-derived signals integrate into stress regulatory networks and may guide the development of novel strategies to prevent or treat stress-related disorders (e.g., anxiety, depression, or stress-aggravated cardiac events). By focusing on the emerging OCN–stress axis, our review highlights an expanding perspective on bone as an endocrine organ influencing stress physiology.

## 1 Introduction

### 1.1 Overview of OCN

OCN, a small peptide consisting of 49 amino acid residues, is synthesized and secreted by osteoblasts and serves as a marker of mature bone tissue (Zoch et al., 2016). Initially, OCN gained attention due to its specific binding to hydroxyapatite, playing a crucial role in bone mineralization (Al-Suhaimi and Al-Jafary, 2020). However, recent studies have revealed that OCN also functions as an endocrine factor, exerting regulatory effects on multiple organ systems throughout the body (Smith et al., 2024). After carboxylation, OCN binds to the bone matrix, while its undercarboxylated form enters the circulation to fulfill endocrine functions (Dirckx et al., 2019). Animal experiments have shown that OCN acts through its receptor, G-protein coupled receptor class C group 6 member A (GPRC6A), which is expressed in various peripheral tissues (largely demonstrated in animal models) (Pi et al., 2017). In the central nervous system, OCN crosses the blood-brain barrier (BBB) and may influence cognition, mood, and neuroprotection through its receptor, G-protein coupled receptor 158 (Gpr158) or G-protein coupled receptor 37 (Gpr37) (Oury et al., 2013). Peripherally, OCN regulates energy metabolism, insulin secretion, and male fertility (Dirckx et al., 2019; Oury et al., 2011). Interestingly, some studies suggest a close relationship between OCN and ASR. Given the emerging recognition of bone as an endocrine organ that contributes to homeostasis it is important to investigate how OCN affects the ASR. Here, we present a comprehensive review of current findings on the OCN–ASR axis, integrating recent evidence and identifying knowledge gaps to be addressed in future research.

### 1.2 Definition and mechanisms of ASR

ASR is a complex set of neuroendocrine, metabolic, and behavioral adaptations initiated by an organism in the face of sudden physiological or psychological stressors to maintain homeostasis (Godoy et al., 2018). While this response is beneficial for coping with immediate threats, excessive or prolonged activation can lead to various health problems (McEwen, 2017). The ASR involves the coordinated activation of the hypothalamic-pituitary-adrenal (HPA) axis and the sympathetic-adrenal-medullary (SAM) axis (Bains et al., 2015). Stress signals are transmitted via neural pathways to the paraventricular nucleus (PVN) of the hypothalamus, stimulating the secretion of corticotropin-releasing factor (CRF) and arginine vasopressin (AVP), which in turn prompt the synthesis of adrenocorticotropic hormone (ACTH) in the pituitary gland and the subsequent release of glucocorticoids (GCs) from the adrenal cortex (Herman et al., 2016). GCs bind to widely distributed receptors and regulate immune, metabolic, and cardiovascular functions; however, their dysregulation is associated with numerous pathologies (Cain and Cidlowski, 2017). Activation of the SAM axis triggers the rapid release of catecholamines, such as epinephrine, facilitating the “fight-or-flight” response during stress (Tank and Lee Wong, 2015). The ASR also involves alterations in cytokines and neurotransmitters, but the key molecular mechanisms underlying these changes remain to be fully elucidated (Ch et al., 2005).

## 2 OCN and ASR

### 2.1 Changes in OCN levels under stress

It has been hypothesized that OCN plays an evolutionary role in enabling vertebrates to respond to danger (the “fight-or-flight” response) (Morris, 2019). Consistent with this concept, multiple studies report that OCN levels significantly increase under acute stress (Berger et al., 2019; Smith et al., 2021). During stress, amygdala signaling triggers the release of glutamate, which enters osteoblasts via the transporter protein Glast and competitively inhibits the γ-carboxylation of OCN. This acute inhibition leads to increased levels of uncarboxylated and undercarboxylated OCN that are released into the circulation within minutes (Berger et al., 2019). Berger et al. found that a surge in serum OCN under acute stress contributes to classic ASR features such as a faster heart rate, enhanced breathing efficiency, and increased energy expenditure (Berger et al., 2019).

Stress-induced OCN elevations have been documented in various contexts. For example, a systematic review of 13 exercise trials reported that acute aerobic exercise can elevate total OCN levels in middle-aged adults (Smith et al., 2021). However, the same review noted that older adults (both men and women) tended to show no significant change in OCN after similar exercise bouts (Smith et al., 2021), suggesting age-related differences in OCN responsiveness (It is worth noting that exercise imposes mechanical load on bone; thus, OCN release in these studies might be related to bone strain in addition to neuroendocrine stress.) In a clinical study of severe trauma, burn patients showed significantly elevated serum OCN in the prolonged stress phase (days 7–56 post-injury) (Muschitz et al., 2016). Jürimäe et al. observed that plasma OCN increased after a 1-h rowing exercise in female participants (Jürimäe et al., 2026). Similarly, Kelly et al. reported a significant rise in salivary OCN levels following 9 h of extreme cold-water dive training (Kelly et al., 2022). In a human psychosocial stress test (public speaking with cross-examination), circulating bioactive OCN levels also increased, correlating with an acute rise in heart rate and blood pressure (Berger et al., 2019). Animal studies mirror these findings: in mice, circulating uncarboxylated OCN was about 50% higher after 45 min of restraint stress and about 150% higher after 150 min of electric foot shock (Berger et al., 2019). Exposure of mice to a predator odor (2,4,5-trimethylthiazole, TMT) induced a rapid OCN spike within about 2.5 min (concurrent with the cortisol peak) that remained elevated for at least 3 h (Berger et al., 2019). Rats subjected to acute foot restraint showed a significant increase in plasma OCN levels as well (Patterson-Buckendahl et al., 2007).

On the other hand, certain stress conditions can lower OCN levels. Some studies reported that patients experiencing acute myocardial infarction (AMI) exhibit decreased serum OCN, possibly due to stress-induced hypercortisolemia inhibiting OCN production (Napal et al., 1993; Goliasch et al., 2011; Ekenstam et al., 1988). Tian et al. proposed that OCN is only increased in ASR scenarios that involve skeletal muscle activity, whereas purely psychological stressors (lacking physical movement) may lead to elevated GCs that suppress OCN levels (Tian et al., 2020). In support of this, Patterson-Buckendahl et al. noted that mild psychological stressors reduced plasma OCN in rats, while severe “fight-or-flight” stressors caused a rise in OCN (Patterson-Buckendahl et al., 1995). They speculated that in conditions like AMI, a reduction in OCN might be an adaptive response—by relieving OCN’s inhibition of the vagus nerve, parasympathetic (vagal) activity could increase to counterbalance the high sympathetic tone during extreme stress.

Table 1 (see below) provides a summary of key studies examining changes in OCN under acute stress across different models and conditions.

**TABLE 1:** A summary of key studies examining changes in OCN under acute stress across different models and conditions.

Study (Year)	Model/Subjects	Stressor/Condition	Key findings
Berger et al. (2019)	Mice; Humans	Foot shock, restraint, predator odor (mice); Public speaking stress test (humans)	Acute stress cause circulating OCN in mice rapid rise (50%–150% rise depending on stressor). In humans, bioactive OCN rise under psychosocial stress, correlating with heart rate and blood pressure rise
Smith et al. (2021)	Middle-aged adults vs. older adults (exercise trials)	Single session of aerobic exercise	Middle-aged adults: OCN rise after acute exercise. Older adults: no significant OCN change. (Suggests age differences in OCN response to stress/exercise.)
Muschitz et al. (2016)	32 adult males (clinical)	Severe burn injury (trauma); early vs. prolonged stress phases	Serum OCN significantly rise during prolonged stress phase (days 7–56 post-injury) compared to early phase, indicating sustained OCN elevation in chronic stress recovery
Jürimäe et al. (2016)	13 female rowers	1-h intensive rowing exercise	Plasma OCN significantly rise post-exercise in all participants, demonstrating OCN response to acute physical stress
Kelly et al. (2022)	Military divers (training)	9-h cold water dive training (extreme physical stress)	Salivary OCN levels rise after prolonged cold stress exposure, reflecting OCN release under extended acute stress
Patterson-Buckendahl et al., (2007)	Rats	Acute foot restraint vs. chronic mild stress	Acute severe stressor: plasma OCN rise. Mild stressor: plasma OCN decrease (OCN response varies with stress intensity)
Napal et al. (1993)	Rats	Acute immobilization stress	Serum OCN decrease under acute stress, concurrent with corticosterone rise (early evidence that cortisol may suppress OCN)
Goliasch et al. (2011)	Young adult men (≤40 years)	Acute myocardial infarction (AMI)	Patients with AMI showed significantly decreased OCN levels vs. controls, consistent with stress-related OCN suppression in acute cardiac events
Ekenstam et al. (1988)	Humans	High-dose corticosteroid infusion (pharmacological stress)	Exogenous cortisol administration → rapid decrease serum OCN (demonstrating GC-mediated OCN suppression)
Tian et al. (2020)	Opinion/letter (cardiac context)	Perspective on OCN in acute stress	OCN rise in ASR with skeletal muscle exertion; OCN decrease in purely neurogenic stress (due to GCs). Reinforces differing OCN responses depending on stress type
Diegel et al. (2020); Moriishi and Komori. (2020)	Mice (OCN gene knockouts)	Genetic absence of OCN	Found no significant metabolic or hormonal abnormalities in OCN-null mice (contrasting prior studies). Suggests OCN’s endocrine role may be context-dependent or compensated by other factors

OCN, Osteocalcin; AMI, Acute myocardial infarction; GC, glucocorticoid.

### 2.2 OCN and HPA axis

The HPA axis is a central component of the mammalian stress response. Upon exposure to a stressor, the hypothalamus releases corticotropin-releasing hormone (CRH), which stimulates the anterior pituitary to secrete ACTH. ACTH then acts on the adrenal cortex to promote the synthesis and release of GCs (primarily cortisol in humans and corticosterone in rodents) (Smith and Vale, 2006). GCs have wide-ranging effects that help the organism adapt to the stressor, but chronically high GC levels can be detrimental (Sapolsky et al., 2000).

**FIGURE 1:**
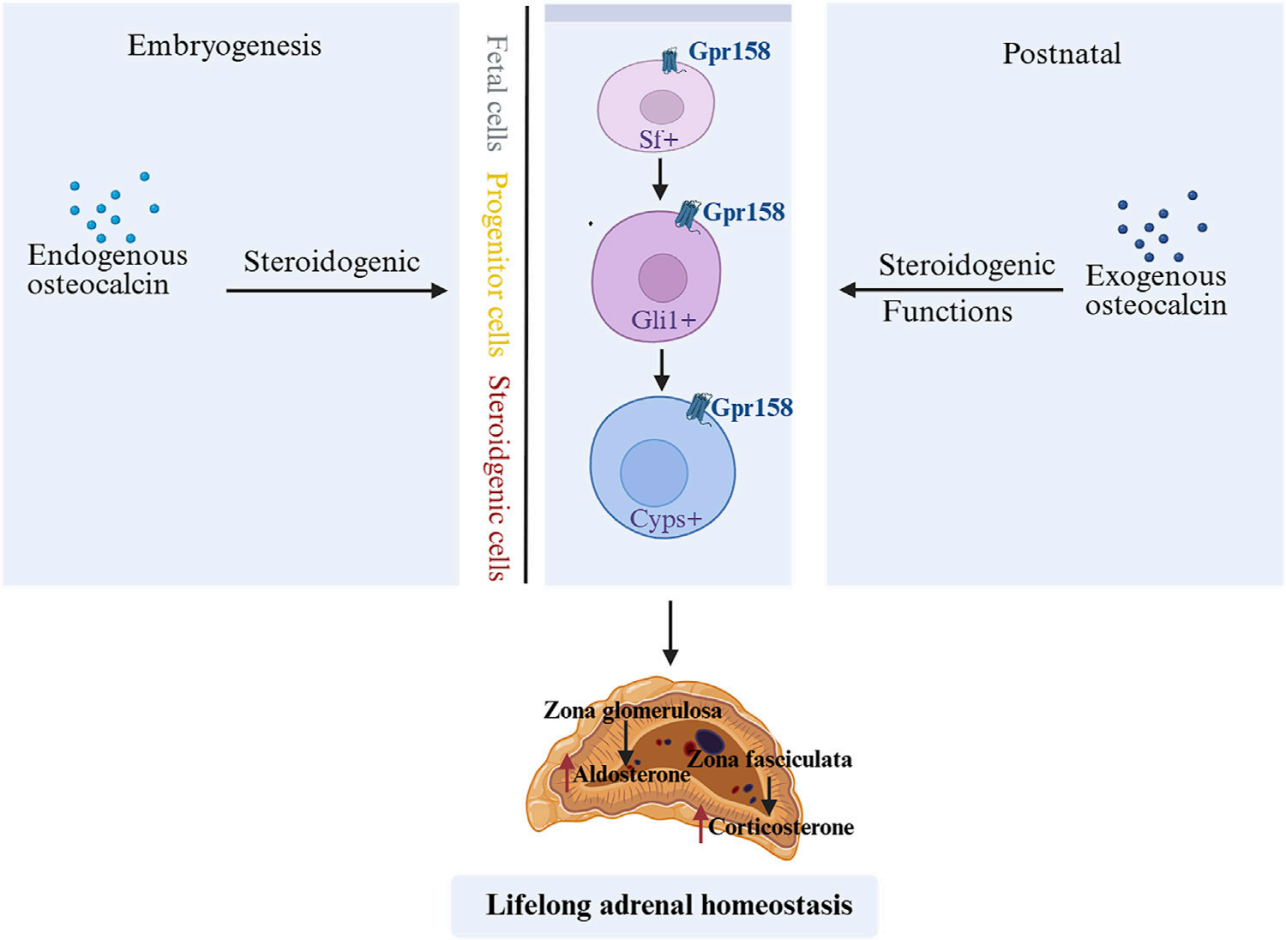
OCN during embryonic and postnatal stages affects lifelong adrenal growth, steroidogenesis, and homeostasis (Yadav et al., 2022). OCN derived from embryos is necessary for the normal expression of Sf1 in fetal adrenal cells and the differentiation of adrenal cells into steroid synthesis cells. It determines the number of steroid synthesis cells present in the adult animal adrenal gland and also regulates the development of the adult animal adrenal gland and the synthesis of steroids in the suprarenal gland. After birth, exogenous OCN can enhance steroidogenesis in rodents and non-human primates. OCN, Osteocalcin; Sf1, steroidogenic factor 1.

**FIGURE 2:**
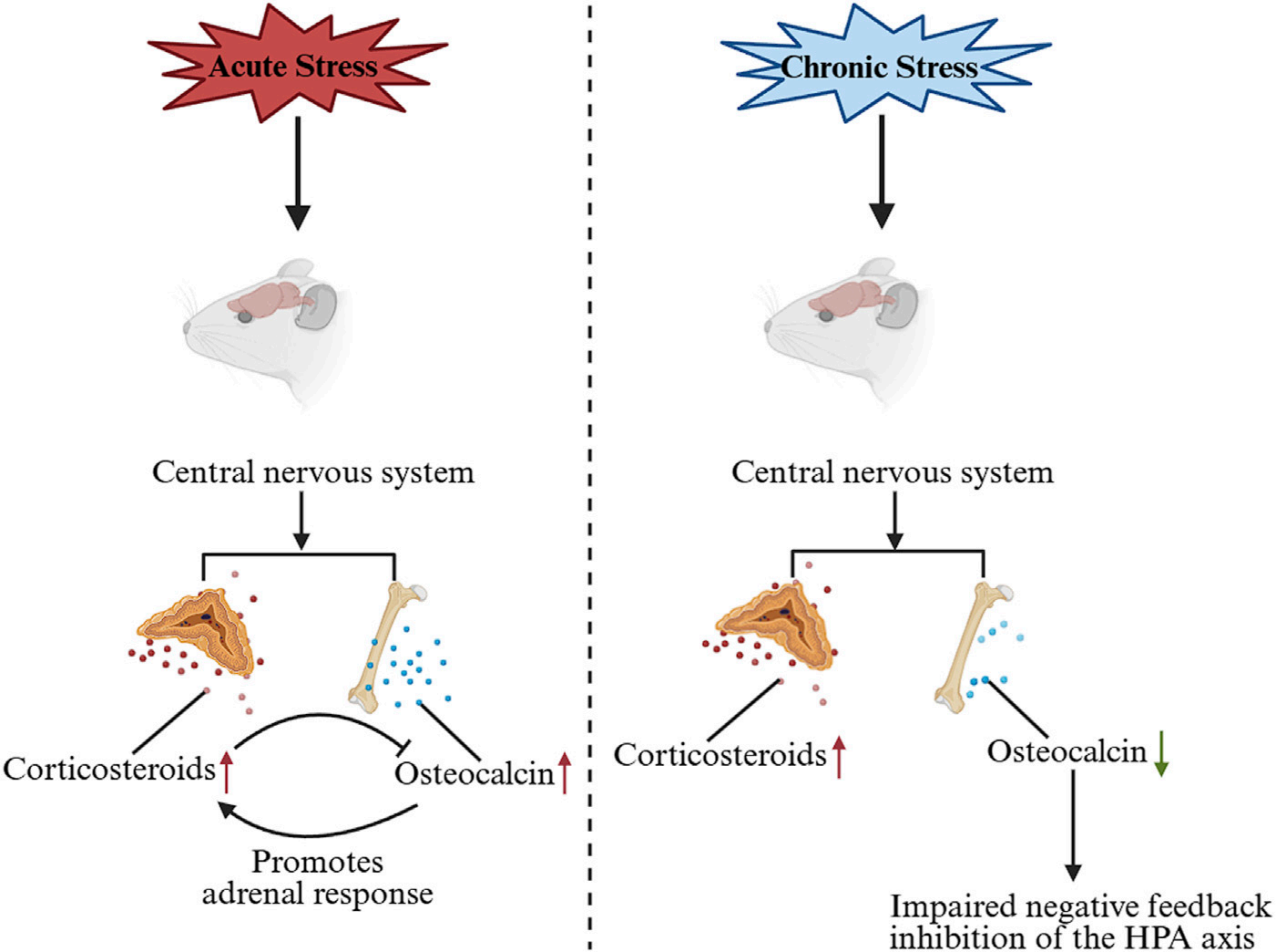
Changes in OCN levels under acute and chronic stress and the effects on the HPA axis. Under acute stress, OCN is secreted in large quantities and promotes the biosynthesis of GC, which inhibits the synthesis and release of OCN. Under chronic stress, OCN levels decrease, leading to impaired negative feedback inhibition of the HPA axis. OCN, Osteocalcin; GC, glucocorticoid.

GCs are known to suppress OCN expression and secretion in both rodents and humans (Di Somma et al., 2003). Consistent with this, adrenalectomy (removal of endogenous GCs production) leads to a dramatic increase in circulating bioactive OCN after stress exposure, compared to sham-operated controls (Berger et al., 2019). Recent studies suggest that OCN, in turn, can influence the HPA-axis stress response. OCN directly promotes the biosynthesis of GCs in rodents and primates. Genetic inactivation of OCN or its receptor significantly impairs adrenal growth and steroidogenesis, indicating that OCN is required for a normal adrenal stress response (Yadav et al., 2022). In fact, OCN from the embryo is necessary for proper expression of steroidogenic factor 1 (Sf1) in fetal adrenal cells and for their differentiation into steroid-producing cells. This developmental role of OCN determines the number of steroidogenic cells present in the adult adrenal gland and impacts adult adrenal growth and GC-producing capacity (Yadav et al., 2022). After birth, exogenous OCN administration can enhance corticosterone/cortisol production in both rodents and non-human primates, an effect that does not depend on upstream HPA signals (Yadav et al., 2022) (Figure 1). Accordingly, OCN knockout mice exhibit blunted HPA axis responses to stress and reduced stress-induced behavioral changes, confirming a regulatory role of OCN in HPA axis activity (Yano et al., 2005). Interestingly, chronic stress (e.g., repeated restraint) causes a decrease in OCN levels in bone and blood in mice, leading to weakened negative feedback on the HPA axis and prolonged cortisol elevation (Patterson-Buckendahl et al., 2012) (Figure 2). Clinically, acute stress-related events such as myocardial ischemia are associated with low OCN and high cortisol levels, supporting the notion that excessive GCs can suppress OCN during stress (Napal et al., 1993; Goliasch et al., 2011; Ekenstam et al., 1988). This disruption of the normal OCN–HPA balance may play a role in stress-related pathology. In summary, the close interplay between OCN and HPA-axis activity provides insight into bone–brain crosstalk in the regulation of stress responses.

### 2.3 OCN and autonomic nervous system

**FIGURE 3:**
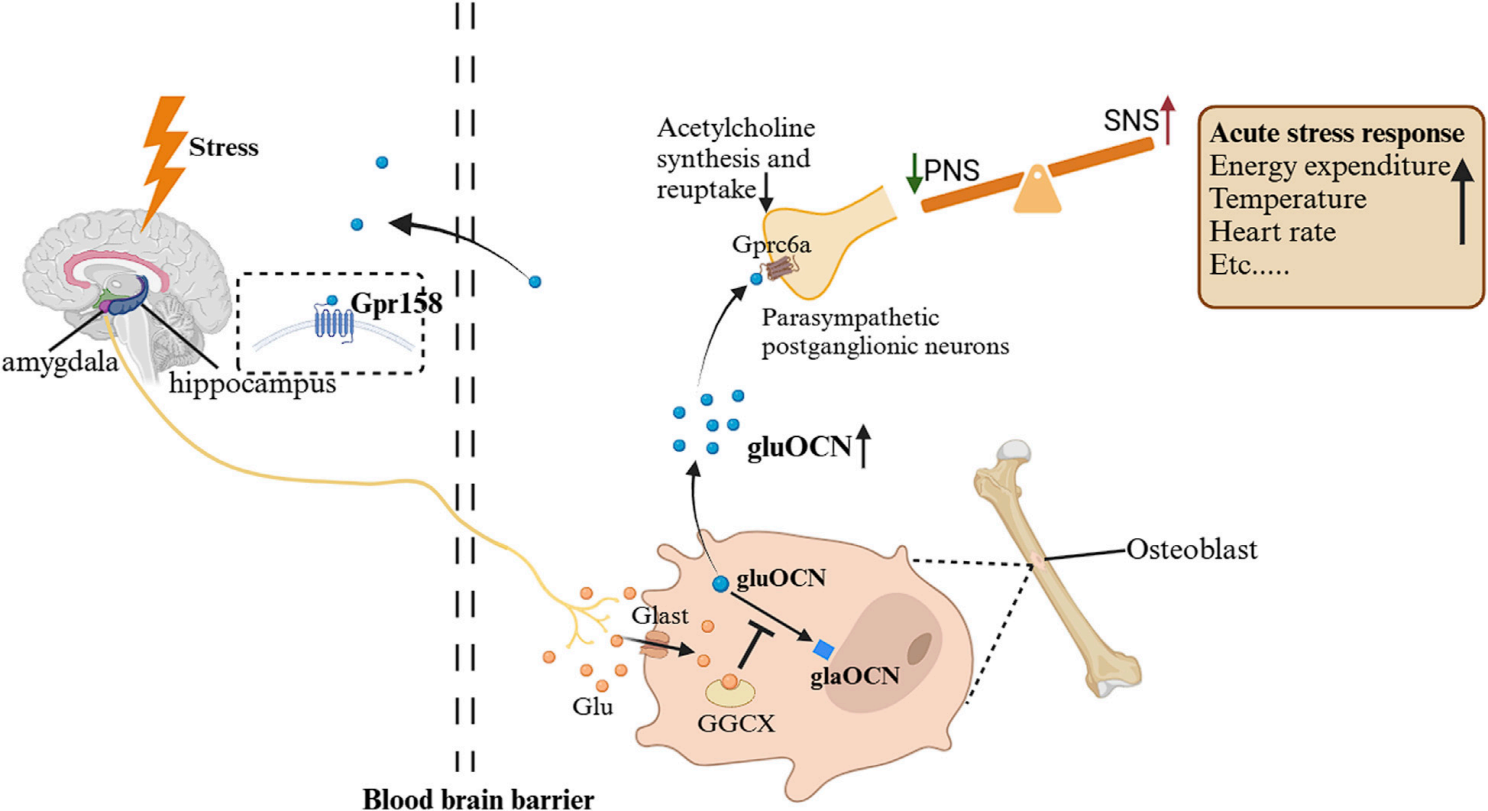
Changes in OCN during ASR and its impact on the autonomic nervous system (Berger and Karsenty, 2022). When exposed to a stressful environment, the stressor sends signals from the basolateral amygdala and other fear centers of the brain, and glutamatergic neurons release the neurotransmitter glutamate, which enters osteoblasts through the transport protein Glast and competitively inhibits GGCX. As a result, levels of uncarboxylated or incompletely carboxylated OCN increase, and uncarboxylated or insufficiently carboxylated OCN can be released into the circulation. They bind to GPRC6A on parasympathetic postganglionic neurons, inhibiting acetylcholine synthesis and reuptake. Therefore, the tension of parasympathetic neurons is inhibited, and the tension of sympathetic neurons is relatively increased, mediating ASR. Gpr158, G protein-coupled receptor 158; GPRC6A, G protein coupled receptor group 6 member a; PNS, parasympathetic nervous system; SNS, sympathetic nervous system; GGCX, gamma-glutamyl carboxylase; ASR, acute stress response.

Acute stress also activates an OCN-mediated pathway affecting the autonomic nervous system. As described above, stress signals from fear centers (such as the basolateral amygdala) (Kondoh et al., 2016) trigger glutamate release, which reaches osteoblasts via Glast and acutely inhibits the gamma-glutamyl carboxylase (GGCX) (Berger et al., 2019). Consequently, bioactive uncarboxylated OCN is rapidly released into the circulation (Berger et al., 2019). OCN then binds to GPRC6A receptors on postganglionic parasympathetic neurons, inhibiting acetylcholine synthesis and release. This reduces parasympathetic (vagal) tone while relatively increasing sympathetic activity, thereby facilitating the full expression of the fight-or-flight response (Berger et al., 2019). Notably, this OCN-driven autonomic effect occurs even in adrenalectomized animals, demonstrating that it operates independently of adrenal catecholamine release (Berger et al., 2019). In essence, OCN acts as a bone-derived signal that acutely tilts the autonomic balance toward sympathetic dominance during stress (Figure 3).

### 2.4 The relationship between OCN and inflammatory responses

Inflammatory responses are an important adaptive mechanism of the body in response to stress, but excessive or prolonged inflammatory reactions can lead to tissue damage and disease onset. Increasing evidence suggests that OCN has anti-inflammatory and immunomodulatory functions. *In-vitro* studies suggested that OCN can inhibit the chemotaxis, activation, and inflammatory factor release of macrophages and neutrophils (Guedes et al., 2018). OCN can enhance the phagocytic ability of macrophages, alleviate inflammation and pain through Gpr37, which is expressed by neurons, oligodendrocytes in the brain and macrophages (Qian et al., 2021; Yang et al., 2016; Szilágyi et al., 2022). In chronic inflammatory disease models, such as rheumatoid arthritis, OCN also exhibits significant anti-inflammatory and tissue-protective effects (Guedes et al., 2018; Liu et al., 2019). Mechanistic studies suggest that OCN may exert anti-inflammatory effects by inhibiting inflammatory signaling pathways such as nuclear factor kappa-B (NF-κB), downregulating the expression of pro-inflammatory cytokines such as tumor necrosis factor-alpha (TNF-α), interleukin-1 beta (IL-1β), and interleukin-6 (IL-6), and promoting the secretion of anti-inflammatory factors such as interleukin-10 (IL-10) and transforming growth factor-beta (TGF-β) (Moser and van der Eerden, 2018; Zhou et al., 2016). Furthermore, some studies suggest that OCN may play a protective role in autoimmune diseases by regulating T cell subset differentiation and function (Mera et al., 2017a; Bonneau et al., 2017). Inflammatory cytokine IL-6 can also regulate the synthesis of OCN. Studies have shown that IL-6 produced by muscles during exercise acts on IL-6 receptors on osteoblasts, promoting the synthesis and secretion of OCN and enhancing exercise ability (Chowdhury et al., 2020).

### 2.5 OCN and stress-induced metabolic adaptations

Acute stress exposure triggers a rapid metabolic shift characterized by increased glucose release, insulin resistance and enhanced lipolysis (Kyrou and Tsigos, 2009). These changes are mediated by the combined actions of GCs and catecholamines released during HPA-axis and sympathetic activation (Nonogaki, 2000).

Given its established role in energy metabolism, OCN may also modulate the metabolic response to acute stress. For instance, uncarboxylated OCN stimulates insulin secretion and improves insulin sensitivity in peripheral tissues (Lee et al., 2007; Ferron et al., 2012). By stimulating insulin release and enhancing insulin action, OCN could counteract the acute stress-induced hyperglycemia and insulin resistance driven by high cortisol and catecholamines. Consistent with this hypothesis, OCN-deficient mice exhibit an exaggerated metabolic response to acute restraint stress: stressed OCN-null mice develop significantly higher blood glucose levels and worse insulin resistance compared to stressed wild-type controls (Berger et al., 2019). The mechanisms by which OCN influences these acute metabolic changes are likely complex and may involve interactions with other hormones. For example, GCs are known to impair insulin secretion from pancreatic β-cells and to reduce insulin sensitivity in peripheral tissues (Di Dalmazi et al., 2012). OCN has also been reported to promote adiponectin secretion from adipocytes (Guedes et al., 2018), an insulin-sensitizing adipokine (Adiponectin levels typically decrease during stress-related sympathetic activation, so OCN-induced adiponectin might help maintain metabolic balance (Luo et al., 2006). In these ways, OCN’s metabolic actions may serve to buffer the adverse metabolic consequences of an acute stress episode.

The relevance of OCN’s metabolic modulation during stress has potential implications for human health. It raises the question of whether boosting OCN activity could mitigate stress-related metabolic disturbances and reduce the risk of stress-exacerbated metabolic diseases. However, most evidence so far is correlational, and further research is needed to establish causality and to determine if OCN’s acute metabolic benefits translate to humans. It will be important to investigate, for example, whether individuals with higher baseline OCN are less prone to stress-induced hyperglycemia or if OCN analogs can blunt metabolic spikes during stress. These inquiries could open new avenues for preventing stress-related metabolic disorders.

## 3 The role of OCN in ASR-related diseases

### 3.1 OCN and anxiety and depression

Anxiety and depression are the most common stress-related mental disorders, severely impacting patients’ quality of life and mental health. Emerging evidence suggests that OCN is involved in the pathogenesis of these mood disorders. (Doom and Gunnar, 2013). Clinical studies have found that peripheral blood total OCN levels are significantly reduced in patients with depression and negatively correlated with symptom severity (Liu et al., 2023; Altindag et al., 2007; Michelson et al., 1996). Uncarboxylated OCN levels are negatively correlated with adolescent depression (Rnic et al., 2024). However, in a study of patients with depression and type 2 diabetes, serum carboxylated OCN levels were positively associated with the severity of the patients’ depression (Nguyen et al., 2020). Another study has shown that there is a positive correlation between plasma total OCN levels and Hamilton scores in patients with depression (Aydin et al., 2011). The reasons for these discrepancies are not yet clear; factors such as age, sex, bone health status, or subtype of depression may play a role. In animal models, OCN-knockout mice exhibit increased anxiety- and depression-like behaviors, whereas administration of exogenous OCN can ameliorate depression-like behaviors induced by chronic stress (Oury et al., 2013; Coburn et al., 2000; Guo et al., 2015; Pedersen, 2017). Mechanistically, OCN is thought to influence brain chemistry: for example, OCN can cross into the brain and has been shown to regulate the metabolism of monoamine neurotransmitters like serotonin and norepinephrine, which are pivotal in mood regulation (Oury et al., 2013; Khrimian et al., 2017). Additionally, OCN may exert anxiolytic and antidepressant effects by multiple interrelated pathways: it can improve HPA-axis feedback (thereby preventing chronic cortisol overload), reduce neuroinflammation, and alleviate oxidative stress in the brain (Dirckx et al., 2019; Levinger et al., 2017; De Toni et al., 2014). Notably, lifestyle interventions known to benefit mood disorders—such as regular physical exercise—also elevate OCN levels (exercise stimulates OCN expression in muscle and bone) (Mera et al., 2017b; Arias-Loste et al., 2014). This correlation raises the intriguing possibility that some beneficial effects of exercise on anxiety and depression are mediated, in part, by OCN. In summary, current evidence indicates that OCN is closely related to the neurobiology of anxiety and depression and could be explored as a novel therapeutic target for these conditions. Nevertheless, more research is needed to fully understand causality and whether modifying OCN levels can directly influence mood in clinical settings.

## 4 Summary and outlook

### 4.1 Current research limitations and gaps

Although encouraging progress has been made in research on the role of OCN in ASR and related diseases, there are still some limitations and gaps in current studies. Firstly, the understanding of the molecular mechanisms by which OCN regulates ASR is still insufficient, especially the dynamic interaction mechanisms between OCN and key ASR signaling pathways such as the HPA axis and autonomic nervous system, which need to be further elucidated (Ning et al., 2022; Mera et al., 2016). Secondly, there is a lack of systematic research on the dose-effect relationship, time window, and sex differences of OCN’s impact on ASR (Mizokami et al., 2014). For example, it is unknown how variations in baseline OCN or administered OCN doses might differentially affect acute stress outcomes, or whether females and males respond differently to OCN during stress. Additionally, many of the observed associations between OCN and stress-related outcomes are correlational, making it difficult to establish causality. For instance, physically active individuals often have lower anxiety and higher OCN levels, but increased OCN due to exercise does not necessarily mean that OCN itself reduces anxiety (other factors like endorphins or improved metabolic health could be at play). Similarly, if a study is conducted in a specific group (e.g., postmenopausal osteoporotic women), the results may not generalize to other populations or age groups. Future studies should account for such confounding factors and include diverse cohorts to clarify OCN’s role across different contexts. Thirdly, acute heart failure (HF) is another common heart disease involving ASR. However, until now, there have been no studies on OCN levels in patients with acute HF. Some studies in patients with chronic HF have shown inconsistent conclusions about changes in OCN levels (Schleithoff et al., 2003; Bozic et al., 2010). It is interesting to study OCN levels and their prognostic value in patients with acute HF, as OCN might reflect heart failure severity and outcomes. However, before OCN can be translated into clinical prevention or treatment strategies, further studies are needed to determine if OCN has a causal role in the observed ASR phenomena and to solidify these associations (e.g., through translational studies and population-level interventions) prior to clinical trials (Oury et al., 2011; Wei et al., 2014). Finally, little is known about the mechanisms of synergisticaction between OCN and other known or unknown bone-derived factors in the regulation of ASR (Li et al., 2016; Ferron and Lacombe, 2014). In-depth exploration of the above issues will help to comprehensively understand the patterns and mechanisms of OCN’s actions in ASR.

### 4.2 Potential applications of OCN in ASR research

Despite the current limitations, OCN and its analogs are expected to become novel targets for the prevention and treatment of abnormal stress responses. Firstly, OCN and its analogs are expected to become novel target for the effective prevention and treatment of stress-related mental disorders such as anxiety and depression (Khrimian et al., 2017; Obri et al., 2018). Secondly, the regulation of individual stress responses using bone-derived factors such as OCN that influence HPA axis activity may become a new approach to improving psychological stress adaptability and preventing stress-related diseases (Kanazawa, 2015; Liu et al., 2016). Thirdly, OCN levels may serve as a novel biomarker for assessing an individual’s susceptibility to acute stress and predicting the risk of stress-related cardiovascular events (Oury et al., 2015; Fernández-Real et al., 2009). In addition, regulating bone-derived factors such as OCN and optimizing the crosstalk between the skeleton and other organs such as the brain may become a systemic strategy for preventing and controlling chronic stress-induced dysfunction of multiple systems, including metabolic and immune systems (Pi and Quarles, 2012; Sabek et al., 2015). Finally, using OCN as an entry point to deeply explore the new mechanisms by which the skeleton, as an endocrine organ, integrates environmental information and dynamically regulates homeostasis, may provide new ideas and targets for translational research on ASR (Oury et al., 2011).

In conclusion, as a representative of bone-derived endocrine factors involved in the regulation of ASR, OCN has opened up new research perspectives for deeply understanding the role of bone-brain, bone-immune, and bone-cardiovascular axis crosstalk in the dynamic regulation of an individual’s stress response. Further elucidating the molecular regulatory mechanisms and pathophysiological significance of OCN and exploring its potential applications in the prevention and treatment of psychosomatic diseases will be important directions for future basic and clinical research. As research in bone endocrinology and psychoneuroendocrinology continues to deepen, breakthroughs in understanding the OCN–ASR axis are likely to emerge. These advances will provide new scientific evidence and strategies for a multi-system approach to stress-related diseases, ultimately contributing to improved physical and mental health.
